# Well-based crystallization of lipidic cubic phase microcrystals for serial X-ray crystallography experiments

**DOI:** 10.1107/S2059798319012695

**Published:** 2019-10-01

**Authors:** Rebecka Andersson, Cecilia Safari, Petra Båth, Robert Bosman, Anastasya Shilova, Peter Dahl, Swagatha Ghosh, Andreas Dunge, Rasmus Kjeldsen-Jensen, Jie Nan, Robert L. Shoeman, Marco Kloos, R. Bruce Doak, Uwe Mueller, Richard Neutze, Gisela Brändén

**Affiliations:** a Department of Chemistry and Molecular Biology, University of Gothenburg, Box 462, SE-405 30 Gothenburg, Sweden; b MAX IV Laboratory, Lund University, Box 118, SE-221 00 Lund, Sweden; c Discovery Sciences, IMED Biotech Unit, AstraZeneca, Pepparedsleden 1, SE-431 50 Gothenburg, Sweden; d Department of Molecular Biology and Genetics, Aarhus University, Gustav Wieds Vej 10C, DK-8000 Aarhus, Denmark; e Max Planck Institute for Medical Research, Jahnstrasse 29, 69120 Heidelberg, Germany

**Keywords:** serial crystallography, lipidic cubic phase, membrane proteins, protein crystallization

## Abstract

Serial crystallography is having an increasing impact on structural biology. This emerging technique opens up new possibilities for studying protein structures at room temperature and investigating structural dynamics using time-resolved X-ray diffraction. A limitation of the method is the intrinsic need for large quantities of well ordered micrometre-sized crystals. Here, a method is presented to screen for conditions that produce microcrystals of membrane proteins in the lipidic cubic phase using a well-based crystallization approach. A key advantage over earlier approaches is that the progress of crystal formation can be easily monitored without interrupting the crystallization process. In addition, the protocol can be scaled up to efficiently produce large quantities of crystals for serial crystallography experiments. Using the well-based crystallization methodology, novel conditions for the growth of showers of microcrystals of three different membrane proteins have been developed. Diffraction data are also presented from the first user serial crystallography experiment performed at MAX IV Laboratory.

## Abstract 2

A novel method is presented to screen for suitable crystallization conditions and produce large amounts of microcrystals of membrane proteins in lipidic cubic phase for serial crystallography experiments.

## 1. Introduction   

### 1.1. Serial crystallography at X-ray free-electron lasers and synchrotrons   

For almost five decades, synchrotron-radiation sources have been the most influential facilities for macromolecular protein crystallography (Dauter *et al.*, 2010 ▸), and cryo-crystallography using single crystals is still the dominant method for solving protein structures at atomic resolution. However, for challenging targets such as membrane proteins, the need for large well ordered crystals can be problematic (Bill *et al.*, 2011 ▸).

X-ray free-electron lasers (XFELs) have transformed the field of structural biology. Taking advantage of the extremely intense ultrashort microfocused X-ray pulses provided by XFELs, serial femtosecond crystallography (SFX) was developed, in which single-shot diffraction patterns are collected from a continuous stream of micrometre-sized crystals at room temperature before they are vaporized (Neutze *et al.*, 2000 ▸; Chapman *et al.*, 2011 ▸). A complete data set is recovered by merging diffraction images collected from thousands of single crystals. An advantage over traditional cryo-crystallography is that the need to grow large crystals is circumvented, and SFX has indeed been successfully used to solve the structures of a number of membrane proteins (Liu *et al.*, 2013 ▸; Kang *et al.*, 2015 ▸; Zhang *et al.*, 2015 ▸; Johansson *et al.*, 2019 ▸). In addition, the problem of X-ray-induced radiation damage can be avoided, which is of specific importance for metal-containing proteins (Nass *et al.*, 2015 ▸; Andersson *et al.*, 2017 ▸). Finally, time-resolved SFX opens up exciting possibilities for capturing rapid protein structural dynamics using a pump–probe approach, which has resulted in several breakthrough studies on light-triggered proteins (Tenboer *et al.*, 2014 ▸; Nango *et al.*, 2016 ▸; Coquelle *et al.*, 2018 ▸; Nogly *et al.*, 2018 ▸).

More recently, the method of serial crystallography has been extended to also include serial synchrotron crystallo­graphy (SSX). This storage-ring-based approach takes advantage of the improved characteristics of the new generation of synchrotrons with microfocused X-ray beams and improved optics, as well as low-noise high-frame-rate detectors and sophisticated software suites (Nogly *et al.*, 2015 ▸; Weinert *et al.*, 2017 ▸). SSX allows data collection on a millisecond time scale and makes the method accessible to a significantly larger number of users.

### 1.2. Crystallization of membrane proteins   

Crystallization of membrane proteins can be very challenging owing to their surface duality, with both hydrophilic and hydrophobic parts, and their instability compared with soluble proteins. A crystallization method that has proven to be successful for many membrane proteins is the lipidic cubic phase (LCP) or *in meso* crystallization technique, in which a lipid–aqueous environment is provided that mimics a bio­logical membrane (Landau & Rosenbusch, 1996 ▸; Cherezov *et al.*, 2002 ▸). The LCP is created by mixing detergent-solubilized protein with a lipid (commonly monoolein) in a glass syringe so that a cubic phase is formed with the protein reconstituted into the lipid bilayer. Crystallization of the protein within the lipidic phase is induced by adding precipitant solution to the LCP. With the use of robot-assisted pipetting of the LCP suspension onto glass sandwich plates in combination with commercial crystallization screens, screening for successful crystallization conditions can be performed quickly using small amounts of protein (Gaisford *et al.*, 2011 ▸). The LCP crystallization method has been especially advantageous for difficult-to-crystallize G-protein coupled receptors (Cherezov *et al.*, 2007 ▸; Zhang *et al.*, 2015 ▸; Johansson *et al.*, 2019 ▸).

### 1.3. Microcrystallization in LCP for serial crystallography experiments   

Although the toothpaste-like consistency of LCP can pose difficulties in the crystallization and handling of crystals, the LCP matrix has turned out to be helpful when delivering microcrystals through a high-viscosity injector in a slow and continuous manner for SFX experiments (Weierstall *et al.*, 2014 ▸; Ishchenko *et al.*, 2016 ▸). Using high-viscosity injectors, sample consumption during an SFX experiment can be reduced significantly compared with the liquid microjets that initially dominated in SFX studies (Chapman *et al.*, 2011 ▸; Schlichting, 2015 ▸). High-viscosity injectors are now used at both XFELs and synchrotron-radiation sources for data collection from protein crystals grown in LCP, as well as crystals grown in aqueous solution, which are mixed with a grease-like delivery medium prior to injection (Kovácsová *et al.*, 2017 ▸). Even when using low-flow-rate high-viscosity injectors, serial crystallography experiments require large amounts of microcrystals, with time-resolved SFX experiments typically demanding several millilitres of microcrystal slurries.

**Figure 1:**
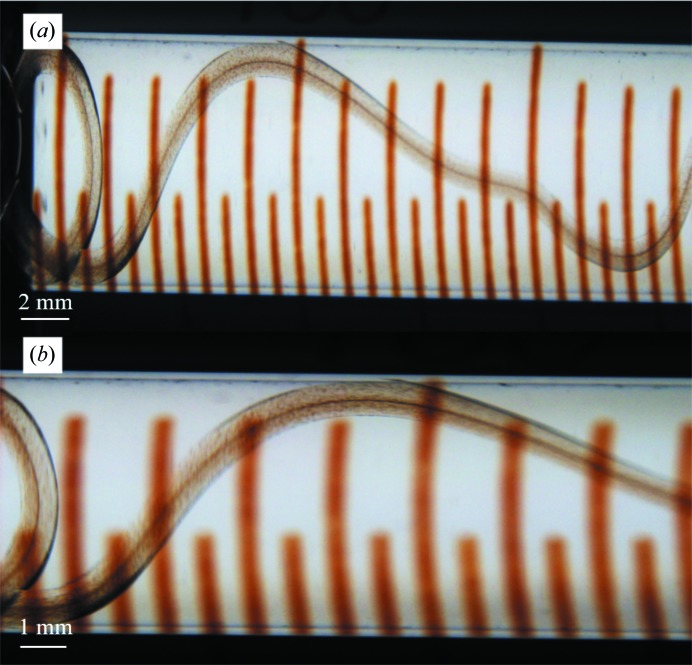
LCP microcrystallization in glass syringes. (*a*) Crystals of *ba*_3_-type cytochrome *c* oxidase visualized at 7.5× magnification in a stereo microscope. (*b*) At a higher magnification of 15, crystals can only be visualized in some parts of the LCP owing to the shape of the syringe and the orientation of the LCP string. Poor focus makes it difficult to determine the crystal quality, shape and size.

The dominant method of producing large amounts of LCP microcrystals has been to use gas-tight glass syringes: typically 60–70 µl precipitant solution is added to a 100 µl syringe, after which 5 µl of LCP suspension is pushed into the precipitant and the syringe is sealed (Liu *et al.*, 2014 ▸; Ishchenko *et al.*, 2016 ▸). To evaluate crystal formation, the syringe is opened and a short string of LCP suspension is applied onto a microscope slide. This has the negative consequence of effectively interrupting the crystallization process. Alternatively, the crystals are visualized directly within the syringe using a stereo microscope. The latter allows the crystals to keep growing, but owing to the thickness of the glass and the shape of the syringe it is difficult to obtain a well focused image at high magnification (Fig. 1 ▸). After successful crystallization, the microcrystal-containing LCP is collected by removing the precipitant solution by slowly pushing the plunger forward and then pooling many batches of crystals from different syringes into one syringe. This method thus requires a large set of glass syringes in which to crystallize the individual LCP batches.

**Figure 2:**
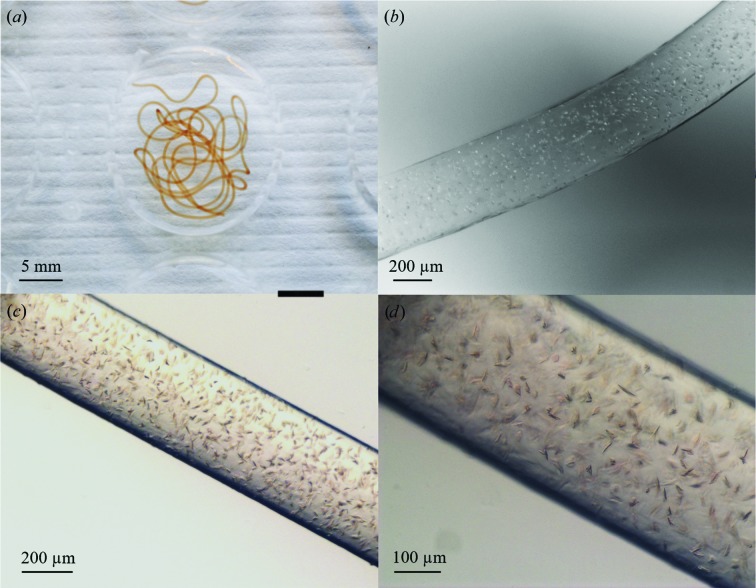
LCP microcrystallization in glass wells. (*a*) A string of LCP suspension (here 40 µl) is dispensed into each well of a nine-well glass plate. Crystals of *ba*_3_-type cytochrome *c* oxidase are visualized in a stereo microscope after three days of incubation at 20°C (*b*) under polarized light at a magnification of 40 and at magnifications of (*c*) 50 and (*d*) 135. Crystal formation can be monitored over time and the crystal quality, shape and density are clearly visible.

Here, we present a well-based crystallization method to screen for successful crystallization conditions and to produce large amounts of microcrystals in LCP to enable serial crystallography experiments of membrane proteins at XFELs and synchrotrons (Fig. 2 ▸). Using the described workflow, novel crystallization conditions for three different integral membrane proteins have been found: *ba*_3_-type cytochrome *c* oxidase (C*c*O), sensory rhodopsin II and a bacterial reaction center. Moreover, X-ray diffraction data extending to 3.6 Å resolution were collected from *ba*_3_-type cytochrome *c* oxidase crystals in the first user SSX experiment performed at the BioMAX beamline at MAX IV Laboratory.

## 2. Procedure for well-based crystallization of lipidic cubic phase microcrystals   

**Figure 3:**
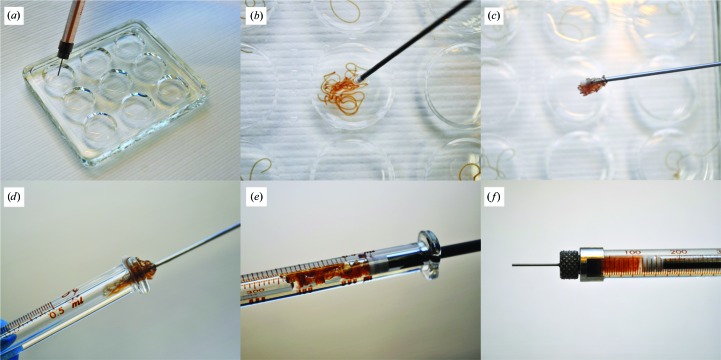
Well-based crystallization procedure. (*a*) A short needle is connected to the syringe to dispense the LCP suspension into the wells of a nine-well glass plate for crystallization. (*b*) The microcrystals are collected by pooling the LCP strings from different wells with a plunger. The LCP blob is transferred (*c*) from the precipitant solution to (*d*) a 500 µl Hamilton syringe from the back. (*e*) A 500 µl plunger is inserted into the syringe and slowly pushed forward to remove any residual precipitant solution. (*f*) A packed syringe of *ba*_3_-type cytochrome *c* oxidase crystals.

Reconstitution of the protein into LCP is achieved using standard protocols, in which the protein is mixed with an appropriate host lipid such as monoolein in a suitable ratio, often between 40% and 50% of aqueous phase for most lipids, in two Hamilton gas-tight 100 µl syringes until the suspension is homogeneous and transparent (Liu *et al.*, 2014 ▸). A nine-well glass plate is prepared with 0.1–1 ml precipitant solution in each of the wells. A short removable needle such as a Mosquito LCP narrow-bore needle is connected to the syringe with the LCP suspension and a string of between 5 and 50 µl of LCP suspension is dispensed into each well [Fig. 3 ▸(*a*)]. The glass plate is then sealed with a ClearVue plastic sheet cut to an appropriate size to fit inside the rim of the plate. This makes it possible to cut open an individual well without disrupting the rest of the plate. During the process of screening for optimal crystallization conditions, it is recommended to start by varying the protein concentration of the LCP and the precipitant concentration in the wells. For this, it is suitable to dispense a small amount (∼5 µl) of LCP per well. Different ratios of LCP to precipitant solution affect the crystallization outcome and should be investigated for each protein. Thus, as a next step, the effect of varying the volume of the crystallization solution in each well, ranging between 0.1 and 1 ml, and the amount of LCP per well, ranging between 5 and 50 µl, should be investigated. This is typically performed in steps of 0.1 ml and 5–10 µl, respectively. If further fine-tuning is needed, the thickness of the LCP string can also be varied by changing the gauge size of the needle that is connected to the LCP-containing syringe.

After crystal formation is complete, the plastic cover is removed and the microcrystals are collected by pooling several LCP strings into one well with the help of a plunger [Fig. 3 ▸(*b*)]. It is important that the amount of time that the LCP is exposed to air is kept to a minimum to prevent dehydration. As long as the LCP is submerged in the well solution, it will not dry out. The larger blob of LCP is then transferred to a 500 µl syringe using the plunger [Figs. 3 ▸(*c*) and 3 ▸(*d*)], a process that only takes a few seconds. The plunger is slowly pushed forward to remove residual precipitant solution [Figs. 3 ▸(*e*) and 3 ▸(*f*)]. The process of collecting crystals from a nine-well glass plate can be completed within minutes. If the crystal-containing LCP has transformed into a more fluid sponge phase, the syringe can be kept in a vertical position with the needle pointing downwards for some minutes to let residual precipitant solution separate from the crystal-containing phase by gravitation before the plunger is pushed forward. Using this method, LCP from the wells of several glass plates can be transferred into the same syringe and large amounts of microcrystals can thus be collected in a simple and efficient way. After the crystals have been packed into an airtight syringe they can typically be stored for weeks without loss of diffraction quality. The nine-well glass plates can be re-used after proper cleaning with detergent and ethanol.

## 3. Materials and methods   

### 3.1. Crystallization materials   

General equipment for LCP crystallization was used: 100 and 500 µl gas-tight Hamilton syringes (Hamilton) to prepare LCP as well as to store and transport microcrystals, ferrules, a heating block for melting the lipid and mixing unions. For crystallization in wells, Mosquito LCP narrow-bore short needles (TTP Labtech), nine-well glass crystallization plates (Hampton Research) for both screening and large-scale production, ClearVue plastic sheets (Molecular Dimensions) to seal the plates and a stereo microscope to monitor the crystal growth over time are needed.

### 3.2. Crystallization of *ba*_3_-type cytochrome *c* oxidase   

*ba*_3_-type cytochrome *c* oxidase (molecular weight 84 kDa) from *Thermus thermophilus* was produced and purified according to a previous report (Andersson *et al.*, 2017 ▸). Freshly purified protein in 5 m*M* HEPES pH 8.0, 0.05%(*w*/*v*) dodecyl-β-d-maltoside (DDM) was concentrated to 0.3 m*M* using an ultrafiltration unit (100 kDa molecular-weight cutoff) and spun down in Eppendorf tubes (1 h, 16 900*g*) to remove any precipitated protein. The protein was then mixed with monoolein [9.9 monoacylglycerol (MAG), Nu-Check Prep; CAS 111-03-5] at a 40:60 protein:lipid ratio in two gas-tight 100 µl syringes as described previously (Caffrey & Porter, 2010 ▸; Liu *et al.*, 2014 ▸). Initial screening for microcrystals was based on a crystallization condition that had previously been used to produce well diffracting large single crystals (Tiefenbrunn *et al.*, 2011 ▸) and was further optimized in nine-well glass plates. The optimization was performed by dispensing up to 20 µl LCP at varying protein concentrations into each well containing 300 µl precipitant solution at varying pH values and concentrations of PEG 400 and NaCl using a Mosquito LCP narrow-bore short needle. The plates were sealed and incubated at 19°C. Well ordered crystals appeared in 37%(*v*/*v*) PEG 400, 1.0–1.4 *M* NaCl, 100 m*M* sodium cacodylate trihydrate pH 5.3 after 2–3 days. The crystal-containing LCP strings were collected and transferred to a 500 µl syringe for storage and transportation to the SPring-8 Angstrom Compact Free Electron Laser (SACLA), Japan. Data collection on the BL3 beamline together with data processing and structure determination have been described in detail elsewhere (Andersson *et al.*, 2017 ▸).

### 3.3. Crystallization of *ba*_3_-type cytochrome *c* oxidase using a nontoxic condition   

*ba*_3_-type C*c*O protein was purified according to a previous report (Andersson *et al.*, 2017 ▸) with the following modifications. The protein was dialyzed once in 5 m*M* HEPES pH 8.0, 0.05%(*w*/*v*) DDM at 4°C overnight and was then loaded onto a HiPrep DEAE FF 16/10 column (GE Healthcare Life Sciences) using 20 m*M* Tris–HCl buffer pH 7.6 with 0.05%(*w*/*v*) DDM. The protein was eluted with 80 m*M* NaCl and concentrated using an ultrafiltration unit (50 kDa molecular-weight cutoff), centrifuged for 20 min at 16 900*g* and loaded onto a Superdex 200 Increase 10/300 GL column (GE Healthcare Life Sciences). Finally, the protein was concentrated to 0.24 m*M* in 20 m*M* Tris–HCl pH 7.6, 0.05%(*w*/*v*) DDM, 80 m*M* NaCl. The purified protein was reconstituted into LCP as described above. Based on the previous microcrystallization condition, an alternative precipitant solution was explored in which the toxic sodium cacodylate trihydrate buffer was exchanged for 2-(*N*-morpholino)ethanesulfonic acid (MES) buffer. The crystallization condition was optimized in glass plates by dispensing LCP-reconstituted protein at different protein concentrations in ∼5 µl aliquots into precipitant solution at varying pH values as well as varying the concentrations of PEG 400 and NaCl. Volumes of precipitant solution ranging between 300 and 800 µl were tested. Large-scale microcrystallization of *ba*_3_-type C*c*O was achieved using a protein concentration of 0.24 m*M* (before mixing with monoolein) and 20 µl LCP strings dispensed in 500 µl 34–37%(*v*/*v*) PEG 400, 1.4 *M* NaCl, 100 m*M* MES pH 5.3 after two days of incubation at room temperature. The crystals were packed into a 500 µl Hamilton syringe and transported to the BioMAX beamline at MAX IV Laboratory for SSX data collection.

### 3.4. Crystallization of sensory rhodopsin II   

Sensory rhodopsin II (SRII; molecular weight 25 kDa) from *Natronomonas pharaonis* was purified according to Hohenfeld *et al.* (1999 ▸) with the following modifications: the detergent *n*-decyl-β-d-maltopyranoside was exchanged for *n*-octyl-β-d-glucopyranoside (β-OG), and a gel-filtration chromatography step was added with buffer consisting of 150 m*M* NaCl, 25 m*M* sodium/potassium phosphate, 0.8%(*w*/*v*) β-OG pH 5.1 after the Ni–NTA chromatography to increase the purity before crystallization. SRII was concentrated to 1.6 m*M* and reconstituted into LCP with monoolein (9.9 MAG) as described above. Initial screening for microcrystals was performed using an LCP Mosquito robot with the MemGold 2 crystallization screen (Newstead *et al.*, 2008 ▸), and the crystals were further optimized using the well-based approach. Protein concentrations of 0.8–1.6 m*M* (before mixing with monoolein) were tested and the concentration of PEG 400 was varied between 30 and 42% in steps of 2%. Finally, the volume of the well solution was varied as described above. The best crystals were obtained using a protein concentration of 1.6 m*M* with 10 µl LCP added to each well containing 400 µl precipitant solution. Large amounts of SRII crystals of 10–40 µm in size were grown at 22°C after 4–12 days in nine-well glass plates in a precipitant solution consisting of 38%(*v*/*v*) PEG 400, 150 m*M* CaCl_2_, 100 m*M* glycine pH 7.5. Microcrystals were tested for diffraction on the BioMAX beamline at MAX IV Laboratory (data not shown).

### 3.5. Crystallization of a bacterial reaction center   

The reaction center from *Blastochloris viridis* (molecular weight 142 kDa) was produced and purified as described previously (Wöhri *et al.*, 2009 ▸; Dods *et al.*, 2017 ▸). For crystallization, thawed protein was centrifuged (16 900*g*, 15 min), concentrated to 0.3 m*M* and mixed with microcrystal seeds (Dods *et al.*, 2017 ▸) at a protein:seed ratio of 100:5. The seeded protein was mixed with monoolein (9.9 MAG) doped with 0.5%(*v*/*v*) ubiquinone-2 in a 40:60 protein:lipid ratio to form the LCP. Initial crystal hits were found by screening around previously published LCP crystallization conditions for the reaction center (Chiu *et al.*, 2000 ▸) using an LCP Mosquito robot. The crystallization conditions were optimized by screening the concentration of 1,2,3-heptanetriol isomer T against the thickness of the LCP string. Following the general well-based crystallization protocol described above, the conditions were then fine-tuned by varying the concentrations of both the protein and the precipitant, as well as the volume ratio between LCP and precipitant solution. Ultimately, 10–12 µl LCP was dispensed into a well with 100 µl precipitant solution consisting of 40 m*M* zinc sulfate, 120 m*M* 1,2,3-heptanetriol isomer T, 100 m*M* sodium citrate pH 6.0 and incubated for three days at room temperature. The reaction-center microcrystals were tested for diffraction on the BL3 beamline at SACLA, Japan (manuscript in preparation).

### 3.6. Data collection at MAX IV Laboratory and data processing   

SSX diffraction data for the *ba*_3_-type C*c*O LCP crystals were collected on the BioMAX macromolecular crystallo­graphy beamline at MAX IV Laboratory in Lund (http://www.maxiv.lu.se/biomax). The viscosity of the LCP phase was fine-tuned by mixing the LCP crystals with monoolein in Hamilton syringes at a crystal:monoolein ratio of 80:15. The crystals were injected using a high-viscosity extrusion injector designed and engineered at MPI Heidelberg by Bruce Doak (manuscript in preparation) with a nozzle diameter of 100 µm and a flow rate of 1.2 µl min^−1^. Data were collected at room temperature using a 5 × 20 µm (FWHM) X-ray beam characterized by a wavelength of 0.98 Å and a flux of 2 × 10^12^ photons s^−1^ (at a ring current of 150 mA). The calculated exposure time per crystal was 0.028 s and the diffraction data were recorded using an EIGER 16M hybrid pixel detector at a frame rate of 100 Hz with the full detector array. 214 170 diffraction images were collected from *ba*_3_-type C*c*O microcrystals and were processed offline using *NanoPeakCell* (Coquelle *et al.*, 2015 ▸), which gave a hit rate of 4.7%. Indexing was performed using *CrystFEL* 0.7.0 (White *et al.*, 2016 ▸), with which 65% of the hits could be indexed. Data extending to a resolution of 3.6 Å were converted to MTZ format, truncated (French & Wilson, 1978 ▸) and phased by molecular replacement with *Phaser* (McCoy *et al.*, 2007 ▸) using PDB entry 5ndc (Andersson *et al.*, 2017 ▸) as a model, after which one round of rigid-body refinement was performed using *REFMAC*5 (Murshudov *et al.*, 2011 ▸), resulting in an *R*_work_ and *R*_free_ of 31.2 and 32.5%, respectively.

The sensory rhodopsin II crystals were tested at BioMAX using the same high-viscosity extrusion injection system with a similar experimental setup and parameters as above. The exposure time per crystal was 0.05 s and data collection was performed with the EIGER detector in 4M mode and a frame rate of 400 Hz.

## 4. Results and discussion   

We present a novel method to screen for optimal conditions and produce large amounts of membrane-protein microcrystals in LCP in nine-well glass plates for serial crystallo­graphy experiments at XFELs and synchrotrons. Compared with the standard procedure of crystallization in glass syringes, this allows easy visualization of the progress of crystallization without interrupting the process. With better focus and higher magnification, crystallization trials can be monitored and evaluated more easily with regard to crystal quality, shape and density (compare Fig. 2 ▸ with Fig. 1 ▸). The well-based system can be scaled up to produce large volumes of LCP microcrystals that can be collected and packed into a Hamilton glass syringe, as shown in Fig. 3 ▸.

### 4.1. Room-temperature structure of *ba_3_*-type cytochrome *c* oxidase   

Cytochrome *c* oxidase is the terminal enzyme of the respiratory chain in mitochondria as well as many bacteria, where it couples the reduction of oxygen to water with proton translocation across a biological membrane. This maintains a proton gradient across the membrane that is used for a variety of energy-requiring processes in the cell (Kaila *et al.*, 2010 ▸). Despite enormous efforts, there is still no complete understanding of how the chemical reaction drives proton pumping. One attractive approach to tackle this question is to use time-resolved serial crystallography to track the conformational changes that occur during the reaction.

The first room-temperature SFX structure of *ba*_3_-type C*c*O (Andersson *et al.*, 2017 ▸) was solved using crystals that were screened and produced using the method described here. Freshly purified *ba*_3_-type C*c*O was concentrated and reconstituted into LCP. Starting conditions that have previously been used to produce large crystals for cryo-crystallography (Tiefenbrunn *et al.*, 2011 ▸) were explored and optimized using a Mosquito crystallization robot to yield well formed microcrystals. In addition to the composition of the precipitant solution, crystal formation is dependent on the volume of both LCP and crystallization buffer, as well as the physical enclosure. It is therefore necessary to optimize the crystallization conditions found in the small-scale sandwich plates when scaling up to produce large amounts of microcrystals. This optimization was performed in nine-well glass plates to best be able to monitor crystal growth. Optimal conditions in which ∼20 µl LCP was dispensed into 300 µl precipitant solution were found after two rounds of optimization. Crystals of *ba*_3_-type C*c*O ranging from 5 to 20 µm in size appeared after 2–3 days of incubation at 20°C. A large batch of crystals was produced in glass plates and collected in a glass syringe, as shown in Fig. 3 ▸. X-ray diffraction data were collected at the Japanese XFEL SACLA using a high-viscosity injector, and the room-temperature SFX structure of *ba*_3_-type C*c*O could be solved to a resolution of 2.3 Å (PDB entry 5ndc), as previously reported (Andersson *et al.*, 2017 ▸). During this experiment, diffraction data from crystals produced according to the well-based procedure were compared with data from crystals produced following the traditional method using syringes, with no discernible difference in quality.

### 4.2. Nontoxic crystallization conditions for microcrystals of *ba_3_*-type cytochrome *c* oxidase   

For SFX experiments, the presence of any toxic compound in the sample adds an additional complication as special care has to be taken to collect the sample after exposure to the X-ray pulse by using a closed injection chamber. To circumvent the need to use the highly toxic compound sodium cacodylate trihydrate, which contains arsenic, in the *ba*_3_-type C*c*O crystallization solution, alternative conditions containing a nontoxic buffer were explored. The well-based method was used to find the optimal condition with regard to the concentrations of salt and precipitant as well as pH for the batch production of microcrystals. From this, a new condition was found after three rounds of optimization in which the sodium cacodylate trihydrate buffer was replaced by MES at pH 5.3 (Fig. 2 ▸). Using this condition, large amounts of microcrystals of 5–20 µm in size were produced for serial crystallography experiments at the Japanese XFEL SACLA (data not shown) and MAX IV Laboratory.

### 4.3. Serial synchrotron crystallography data collection from C*c*O microcrystals at MAX IV Laboratory   

**Figure 4:**
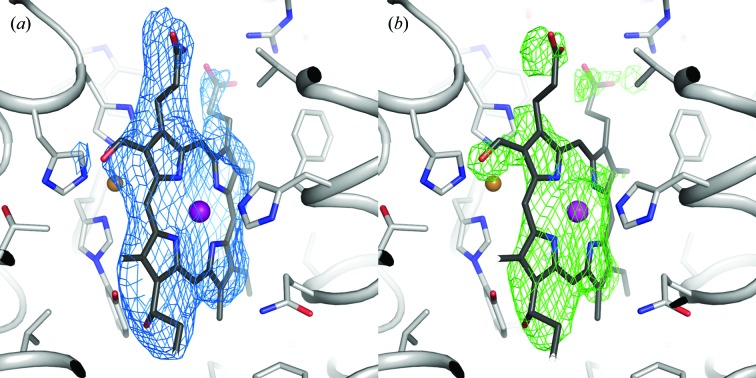
Cytochrome *c* oxidase electron-density maps. Electron-density maps calculated from the BioMAX C*c*O data extending to 3.6 Å resolution. (*a*) 2*F*_o_ − *F*_c_ electron-density map contoured at 1.0σ around haem *a*_3_. (*b*) OMIT map calculated around haem *a*_3_ using *phenix.polder* (Liebschner *et al.*, 2017 ▸) contoured at 3.0σ around haem *a*_3_.

X-ray diffraction data were collected from *ba*_3_-type C*c*O microcrystals on the BioMAX beamline as part of the first user serial crystallography experiment to be conducted at MAX IV Laboratory. The LCP-grown crystals were injected across the X-ray beam using a high-viscosity extrusion injector designed and engineered at MPI Heidelberg by Bruce Doak (manuscript in preparation), and diffraction data were collected on an EIGER 16M hybrid pixel detector. A complete data set extending to 3.6 Å resolution was obtained from 215 000 collected images, of which 6513 could be indexed (Table 1 ▸). The data-processing statistics indicate a good-quality data set, although of limited resolution. As is typical for serial crystallography data, we have a rather high multiplicity of 18. The resolution of 3.6 Å can be compared with previously obtained data from SACLA, which resulted in a 2.3 Å resolution structure (Andersson *et al.*, 2017 ▸). This may be owing in part to differences between the effective photon flux per image as well as a difference in the X-ray scattering cross-section when comparing the BioMAX and SACLA experiments. Specific reasons are the differences in number of X-ray photons per exposure (2 × 10^10^ at BioMAX versus 8.5 × 10^10^ at SACLA) and elastic scattering cross-sections (data collected at 12.7 keV at BioMAX and at 7.6 keV at SACLA), as well as the fact that the X-ray beam at BioMAX (5 × 20 µm) was on average larger than a projection of the crystal that it interacted with (crystal size ∼5–20 µm for both experiments), which was not an issue at SACLA (X-ray beam 1.3 × 1.5 µm). We estimate that these combined effects lead to the scattering per crystal at BioMAX being approximately 2% of that at SACLA. Another difference is that a nozzle diameter of 75 µm was used at SACLA, whereas the BioMAX data were collected using a 100 µm nozzle, leading to higher background scattering from the LCP in the BioMAX experiment. However, as this was the first user SSX experiment at BioMAX, there is the potential for improved results in the future by optimization of both the crystal-injection and data-collection parameters. The 2*F*_o_ − *F*_c_ electron-density map calculated from the BioMAX data after one round of rigid-body refinement is shown in Fig. 4 ▸(*a*) and the OMIT map density around the haem *a*_3_ cofactor is shown in Fig. 4 ▸(*b*).

### 4.4. Well diffracting microcrystals of sensory rhodopsin II   

**Figure 5:**
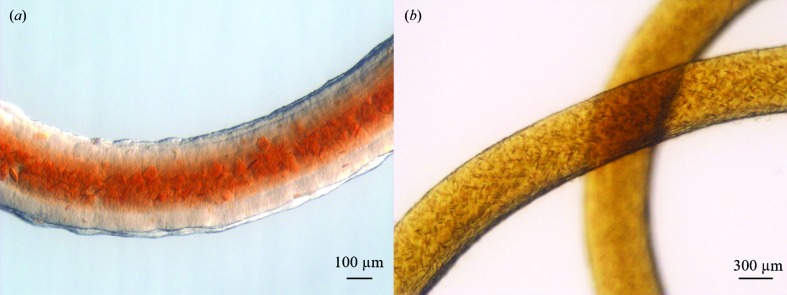
LCP microcrystals of other target proteins produced in wells. (*a*) SRII microcrystals 10–40 µm in size produced without purple membrane that diffract to 2.7 Å resolution at a synchrotron. (*b*) Microcrystals of bacterial reaction center of around 50 µm in size that diffract to 2.4 Å resolution at an XFEL.

SRII is a signaling membrane protein that is responsible for negative phototaxis in haloarchaea. It is often found in complex with its signal transducer HtrII and has served as a model system for signal transduction across the membrane in bacteria (Spudich & Luecke, 2002 ▸; Royant *et al.*, 2001 ▸). Using the well-based crystallization method, a new crystallization protocol for *N. pharaonis* SRII microcrystals was developed after four rounds of optimization. The new protocol does not require reconstitution into purple membrane lipids, as opposed to previous protocols (Luecke *et al.*, 2001 ▸). The microcrystals [Fig. 5 ▸(*a*)] showed diffraction to ∼2.7 Å resolution when tested at the BioMAX beamline at MAX IV Laboratory using the high-viscosity extrusion injector serial crystallography setup as described above (data not shown). The new crystal form shows promise for future time-resolved experiments.

### 4.5. Well diffracting microcrystals of a bacterial reaction center   

The first membrane-protein structure to be solved by X-ray crystallography was that of the reaction center, an analog of the protein photosystem II, from the photosynthetic bacterium *B. viridis* (Deisenhofer *et al.*, 1984 ▸, 1985 ▸; Martin *et al.*, 2018 ▸). Since then it has served as a model system for the study of photosynthetic reactions. Previous SFX experiments have been carried out with reaction-center crystals produced in lipidic sponge phase (Johansson *et al.*, 2013 ▸), a swollen analog of LCP, and by the detergent-based vapor-diffusion method (Dods *et al.*, 2017 ▸). However, these crystals need to be injected using a liquid-based injector such as the gas dynamic virtual nozzle system, and are thus not suitable for experiments at synchrotrons, where a slower flow rate is required. With the aim of finding a novel crystallization condition to produce microcrystals of the reaction center in LCP for time-resolved SFX experiments, well-based crystallization was used as the screening method. Protein spiked with crystals seeds grown using the vapor-diffusion crystallization protocol (Dods *et al.*, 2017 ▸) was reconstituted into LCP and successfully produced crystals. The success of this approach is particularly interesting as the space group of the crystals used to produce the seeds differs from that of the resulting LCP crystals. Moreover, the seeding step is required for successful LCP crystallization under our conditions (manuscript in preparation). Five rounds of optimization of the crystallization protocol were required to find optimal conditions. The best microcrystals, of 20–50 µm in size, were grown using a small volume of 100 µl precipitant solution consisting of 40 m*M* zinc sulfate, 120 m*M* 1,2,3-heptanetriol isomer T, 100 m*M* sodium citrate pH 6.0 in each well of the nine-well glass plate [Fig. 5 ▸(*b*)]. The crystals were tested on the BL3 beamline at SACLA, where they diffracted to 2.4 Å resolution (manuscript in preparation).

## 5. Conclusions   

The development of serial crystallography at XFELs and synchrotrons has opened up completely new possibilities within structural biology, as we can study protein structures at room temperature and investigate structural dynamics using time-resolved experiments. A severe limitation for these experiments is the need for large amounts of well ordered microcrystals. Membrane proteins are in many cases particularly difficult to crystallize. For a number of membrane proteins, high-resolution crystal structures have successfully been solved from crystals formed using LCP-reconstituted protein (Cherezov *et al.*, 2007 ▸; Zhang *et al.*, 2015 ▸; Johansson *et al.*, 2019 ▸). As an added advantage for serial experiments, the LCP matrix provides a suitable carrier medium for slow-running high-viscosity injectors. There is, however, a need to simplify the process of producing large amounts of LCP microcrystals for serial crystallography experiments.

Here, we present a method to screen for suitable crystallization conditions for microcrystals in LCP. Using nine-well glass plates, the progress of crystal formation can easily be monitored without interrupting the crystallization process. In addition, the method is suitable for scaling up to produce large amounts of microcrystals, as the crystal-containing LCP in the wells can readily be pooled, picked up and packed into a glass syringe. We also foresee that this method may be useful to introduce various chemicals or ligands such as small-molecule drug compounds to the protein after crystal formation through the addition of the chemical/ligand to the LCP-containing well solution. Solving protein–ligand complex structures frequently requires screening different ligand concentrations and testing various solvents to avoid disrupting the crystals upon addition of the ligand. Our method allows screening for optimal conditions for complex formation with limited amounts of LCP sample, something that is not easily achievable using the traditional methodology of LCP microcrystallization in glass syringes (Liu *et al.*, 2014 ▸).

The usefulness of well-based crystallization is showcased by the first room-temperature structure of *ba*_3_-type C*c*O, where the method was applied to optimize the crystallization conditions as well as for large-scale batch production of microcrystals for an XFEL serial crystallography experiment (Andersson *et al.*, 2017 ▸).

We further exemplify the method by showing three cases in which novel crystallization conditions have been developed to produce microcrystals of the integral membrane proteins *ba*_3_-type cytochrome *c* oxidase, sensory rhodopsin II and bacterial reaction center, all resulting in well diffracting crystals suitable for serial crystallography experiments.

Finally, we describe the first user serial crystallography experiment at MAX IV Laboratory. A complete X-ray diffraction data set for *ba*_3_-type C*c*O extending beyond 3.6 Å resolution was collected using the high-viscosity extrusion injector at the BioMAX beamline. This opens up very exciting possibilities for performing SSX experiments at the Swedish synchrotron even before the serial crystallography beamline, MicroMAX, becomes operational.

## Figures and Tables

**Table 1:** Crystallographic data and processing statistics Serial crystallography data for *ba*_3_-type cytochrome *c* oxidase collected at BioMAX at MAX IV Laboratory.

Temperature (K)	298
Exposure time (s)	0.028
Space group	*C*121
*a*, *b*, *c* (Å)	145.17, 100.15, 96.64
α, β, γ (°)	90.0, 126.64, 90.0
No. of collected images	214170
Average hit rate (%)	4.7
No. of indexed images	6513
Indexing rate (%)	64.7
Total No. of reflections	505407
No. of unique reflections	27514
Multiplicity	18
Completeness (%)	100 (100)
〈*I*/σ(*I*)〉	3 (1.3)
Resolution range (Å)	77.5–3.6 (3.66–3.60)
*R*_split_	0.31 (0.85)
CC*	0.89 (0.52)
*R*_work_/*R*_free_ (%)	31.2/32.5

## References

[bb1] Andersson, R., Safari, C., Dods, R., Nango, E., Tanaka, R., Yamashita, A., Nakane, T., Tono, K., Joti, Y., Båth, P., Dunevall, E., Bosman, R., Nureki, O., Iwata, S., Neutze, R. & Brändén, G. (2017). *Sci. Rep.* **7**, 4518. 10.1038/s41598-017-04817-z PMC5495810 28674417

[bb2] Bill, R. M., Henderson, P. J., Iwata, S., Kunji, E. R., Michel, H., Neutze, R., Newstead, S., Poolman, B., Tate, C. G. & Vogel, H. (2011). *Nature Biotechnol.* **29**, 335–340. 10.1038/nbt.1833 21478852

[bb3] Caffrey, M. & Porter, C. (2010). *J. Vis. Exp.*, 1712. 10.3791/1712 PMC3144658 21113125

[bb4] Chapman, H. N., Fromme, P., Barty, A., White, T. A., Kirian, R. A., Aquila, A., Hunter, M. S., Schulz, J., DePonte, D. P., Weierstall, U., Doak, R. B., Maia, F. R. N. C., Martin, A. V., Schlichting, I., Lomb, L., Coppola, N., Shoeman, R. L., Epp, S. W., Hartmann, R., Rolles, D., Rudenko, A., Foucar, L., Kimmel, N., Weidenspointner, G., Holl, P., Liang, M., Barthelmess, M., Caleman, C., Boutet, S., Bogan, M. J., Krzywinski, J., Bostedt, C., Bajt, S., Gumprecht, L., Rudek, B., Erk, B., Schmidt, C., Hömke, A., Reich, C., Pietschner, D., Strüder, L., Hauser, G., Gorke, H., Ullrich, J., Herrmann, S., Schaller, G., Schopper, F., Soltau, H., Kühnel, K.-U., Messer­schmidt, M., Bozek, J. D., Hau-Riege, S. P., Frank, M., Hampton, C. Y., Sierra, R. G., Starodub, D., Williams, G. J., Hajdu, J., Timneanu, N., Seibert, M. M., Andreasson, J., Rocker, A., Jönsson, O., Svenda, M., Stern, S., Nass, K., Andritschke, R., Schröter, C.-D., Krasniqi, F., Bott, M., Schmidt, K. E., Wang, X., Grotjohann, I., Holton, J. M., Barends, T. R. M., Neutze, R., Marchesini, S., Fromme, R., Schorb, S., Rupp, D., Adolph, M., Gorkhover, T., Andersson, I., Hirsemann, H., Potdevin, G., Graafsma, H., Nilsson, B. & Spence, J. C. H. (2011). *Nature (London)*, **470**, 73–77.

[bb5] Cherezov, V., Clogston, J., Misquitta, Y., Abdel-Gawad, W. & Caffrey, M. (2002). *Biophys. J.* **83**, 3393–3407. 10.1016/S0006-3495(02)75339-3 PMC1302414 12496106

[bb6] Cherezov, V., Rosenbaum, D. M., Hanson, M. A., Rasmussen, S. G. F., Thian, F. S., Kobilka, T. S., Choi, H.-J., Kuhn, P., Weis, W. I., Kobilka, B. K. & Stevens, R. C. (2007). *Science*, **318**, 1258–1265. 10.1126/science.1150577 PMC2583103 17962520

[bb7] Chiu, M. L., Nollert, P., Loewen, M. C., Belrhali, H., Pebay-Peyroula, E., Rosenbusch, J. P. & Landau, E. M. (2000). *Acta Cryst.* D**56**, 781–784. 10.1107/s0907444900004716 10818364

[bb8] Coquelle, N., Brewster, A. S., Kapp, U., Shilova, A., Weinhausen, B., Burghammer, M. & Colletier, J.-P. (2015). *Acta Cryst.* D**71**, 1184–1196. 10.1107/S1399004715004514 PMC4427202 25945583

[bb9] Coquelle, N., Sliwa, M., Woodhouse, J., Schirò, G., Adam, V., Aquila, A., Barends, T. R. M., Boutet, S., Byrdin, M., Carbajo, S., De la Mora, E., Doak, R. B., Feliks, M., Fieschi, F., Foucar, L., Guillon, V., Hilpert, M., Hunter, M. S., Jakobs, S., Koglin, J. E., Kovacsova, G., Lane, T. J., Lévy, B., Liang, M., Nass, K., Ridard, J., Robinson, J. S., Roome, C. M., Ruckebusch, C., Seaberg, M., Thepaut, M., Cammarata, M., Demachy, I., Field, M., Shoeman, R. L., Bourgeois, D., Colletier, J.-P., Schlichting, I. & Weik, M. (2018). *Nature Chem.* **10**, 31–37. 10.1038/nchem.2853 29256511

[bb10] Dauter, Z., Jaskolski, M. & Wlodawer, A. (2010). *J. Synchrotron Rad.* **17**, 433–444. 10.1107/S0909049510011611 PMC3089015 20567074

[bb11] Deisenhofer, J., Epp, O., Miki, K., Huber, R. & Michel, H. (1984). *J. Mol. Biol.* **180**, 385–398. 10.1016/s0022-2836(84)80011-x 6392571

[bb12] Deisenhofer, J., Epp, O., Miki, K., Huber, R. & Michel, H. (1985). *Nature (London)*, **318**, 618–624. 10.1038/318618a0 22439175

[bb13] Dods, R., Båth, P., Arnlund, D., Beyerlein, K. R., Nelson, G., Liang, M., Harimoorthy, R., Berntsen, P., Malmerberg, E., Johansson, L., Andersson, R., Bosman, R., Carbajo, S., Claesson, E., Conrad, C. E., Dahl, P., Hammarin, G., Hunter, M. S., Li, C., Lisova, S., Milathianaki, D., Robinson, J., Safari, C., Sharma, A., Williams, G., Wickstrand, C., Yefanov, O., Davidsson, J., DePonte, D. P., Barty, A., Brändén, G. & Neutze, R. (2017). *Structure*, **25**, 1461–1468. 10.1016/j.str.2017.07.002 28781082

[bb14] French, S. & Wilson, K. (1978). *Acta Cryst.* A**34**, 517–525.

[bb15] Gaisford, W., Schertler, G. & Edwards, P. (2011). *Nature Methods*, **8**, 520.

[bb16] Hohenfeld, I. P., Wegener, A. A. & Engelhard, M. (1999). *FEBS Lett.* **442**, 198–202. 10.1016/s0014-5793(98)01659-7 9929001

[bb17] Ishchenko, A., Cherezov, V. & Liu, W. (2016). *J. Vis. Exp.*, e54463. 10.3791/54463 PMC5092055 27683972

[bb18] Johansson, L. C., Arnlund, D., Katona, G., White, T. A., Barty, A., DePonte, D. P., Shoeman, R. L., Wickstrand, C., Sharma, A., Williams, G. J., Aquila, A., Bogan, M. J., Caleman, C., Davidsson, J., Doak, R. B., Frank, M., Fromme, R., Galli, L., Grotjohann, I., Hunter, M. S., Kassemeyer, S., Kirian, R. A., Kupitz, C., Liang, M., Lomb, L., Malmerberg, E., Martin, A. V., Messerschmidt, M., Nass, K., Redecke, L., Seibert, M. M., Sjöhamn, J., Steinbrener, J., Stellato, F., Wang, D., Wahlgren, W. Y., Weierstall, U., Westenhoff, S., Zatsepin, N. A., Boutet, S., Spence, J. C. H., Schlichting, I., Chapman, H. N., Fromme, P. & Neutze, R. (2013). *Nature Commun.* **4**, 2911. 10.1038/ncomms3911 PMC3905732 24352554

[bb19] Johansson, L. C., Stauch, B., McCorvy, J. D., Han, G. W., Patel, N., Huang, X.-P., Batyuk, A., Gati, C., Slocum, S. T., Li, C., Grandner, J. M., Hao, S., Olsen, R. H. J., Tribo, A. R., Zaare, S., Zhu, L., Zatsepin, N. A., Weierstall, U., Yous, S., Stevens, R. C., Liu, W., Roth, B. L., Katritch, V. & Cherezov, V. (2019). *Nature (London)*, **569**, 289–292. 10.1038/s41586-019-1144-0 PMC6589158 31019305

[bb20] Kaila, V. R., Verkhovsky, M. I. & Wikström, M. (2010). *Chem. Rev.* **110**, 7062–7081. 10.1021/cr1002003 21053971

[bb21] Kang, Y., Zhou, X. E., Gao, X., He, Y., Liu, W., Ishchenko, A., Barty, A., White, T. A., Yefanov, O., Han, G. W., Xu, Q., de Waal, P. W., Ke, J., Tan, M. H. E., Zhang, C., Moeller, A., West, G. M., Pascal, B. D., Van Eps, N., Caro, L. N., Vishnivetskiy, S. A., Lee, R. J., Suino-Powell, K. M., Gu, X., Pal, K., Ma, J., Zhi, X., Boutet, S., Williams, G. J., Messerschmidt, M., Gati, C., Zatsepin, N. A., Wang, D., James, D., Basu, S., Roy-Chowdhury, S., Conrad, C. E., Coe, J., Liu, H., Lisova, S., Kupitz, C., Grotjohann, I., Fromme, R., Jiang, Y., Tan, M., Yang, H., Li, J., Wang, M., Zheng, Z., Li, D., Howe, N., Zhao, Y., Standfuss, J., Diederichs, K., Dong, Y., Potter, C. S., Carragher, B., Caffrey, M., Jiang, H., Chapman, H. N., Spence, J. C. H., Fromme, P., Weierstall, U., Ernst, O. P., Katritch, V., Gurevich, V. V., Griffin, P. R., Hubbell, W. L., Stevens, R. C., Cherezov, V., Melcher, K. & Xu, H. E. (2015). *Nature (London)*, **523**, 561–567.

[bb22] Kovácsová, G., Grünbein, M. L., Kloos, M., Barends, T. R. M., Schlesinger, R., Heberle, J., Kabsch, W., Shoeman, R. L., Doak, R. B. & Schlichting, I. (2017). *IUCrJ*, **4**, 400–410. 10.1107/S2052252517005140 PMC5571803 28875027

[bb23] Landau, E. M. & Rosenbusch, J. P. (1996). *Proc. Natl Acad. Sci. USA*, **93**, 14532–14535. 10.1073/pnas.93.25.14532 PMC26167 8962086

[bb24] Liebschner, D., Afonine, P. V., Moriarty, N. W., Poon, B. K., Sobolev, O. V., Terwilliger, T. C. & Adams, P. D. (2017). *Acta Cryst.* D**73**, 148–157. 10.1107/S2059798316018210 PMC5297918 28177311

[bb25] Liu, W., Ishchenko, A. & Cherezov, V. (2014). *Nature Protoc.* **9**, 2123–2134. 10.1038/nprot.2014.141 PMC4209290 25122522

[bb26] Liu, W., Wacker, D., Gati, C., Han, G. W., James, D., Wang, D., Nelson, G., Weierstall, U., Katritch, V., Barty, A., Zatsepin, N. A., Li, D., Messerschmidt, M., Boutet, S., Williams, G. J., Koglin, J. E., Seibert, M. M., Wang, C., Shah, S. T., Basu, S., Fromme, R., Kupitz, C., Rendek, K. N., Grotjohann, I., Fromme, P., Kirian, R. A., Beyerlein, K. R., White, T. A., Chapman, H. N., Caffrey, M., Spence, J. C. H., Stevens, R. C. & Cherezov, V. (2013). *Science*, **342**, 1521–1524. 10.1126/science.1244142 PMC3902108 24357322

[bb27] Luecke, H., Schobert, B., Lanyi, J. K., Spudich, E. N. & Spudich, J. L. (2001). *Science*, **293**, 1499–1503. 10.1126/science.1062977 PMC4996266 11452084

[bb28] Martin, W. F., Bryant, D. A. & Beatty, J. T. (2018). *FEMS Microbiol. Rev.* **42**, 205–231. 10.1093/femsre/fux056 PMC5972617 29177446

[bb29] McCoy, A. J., Grosse-Kunstleve, R. W., Adams, P. D., Winn, M. D., Storoni, L. C. & Read, R. J. (2007). *J. Appl. Cryst.* **40**, 658–674. 10.1107/S0021889807021206 PMC2483472 19461840

[bb30] Murshudov, G. N., Skubák, P., Lebedev, A. A., Pannu, N. S., Steiner, R. A., Nicholls, R. A., Winn, M. D., Long, F. & Vagin, A. A. (2011). *Acta Cryst.* D**67**, 355–367. 10.1107/S0907444911001314 PMC3069751 21460454

[bb31] Nango, E., Royant, A., Kubo, M., Nakane, T., Wickstrand, C., Kimura, T., Tanaka, T., Tono, K., Song, C., Tanaka, R., Arima, T., Yamashita, A., Kobayashi, J., Hosaka, T., Mizohata, E., Nogly, P., Sugahara, M., Nam, D., Nomura, T., Shimamura, T., Im, D., Fujiwara, T., Yamanaka, Y., Jeon, B., Nishizawa, T., Oda, K., Fukuda, M., Andersson, R., Båth, P., Dods, R., Davidsson, J., Matsuoka, S., Kawatake, S., Murata, M., Nureki, O., Owada, S., Kameshima, T., Hatsui, T., Joti, Y., Schertler, G., Yabashi, M., Bondar, A. N., Standfuss, J., Neutze, R. & Iwata, S. (2016). *Science*, **354**, 1552–1557. 10.1126/science.aah3497 28008064

[bb32] Nass, K., Foucar, L., Barends, T. R. M., Hartmann, E., Botha, S., Shoeman, R. L., Doak, R. B., Alonso-Mori, R., Aquila, A., Bajt, S., Barty, A., Bean, R., Beyerlein, K. R., Bublitz, M., Drachmann, N., Gregersen, J., Jönsson, H. O., Kabsch, W., Kassemeyer, S., Koglin, J. E., Krumrey, M., Mattle, D., Messerschmidt, M., Nissen, P., Reinhard, L., Sitsel, O., Sokaras, D., Williams, G. J., Hau-Riege, S., Timneanu, N., Caleman, C., Chapman, H. N., Boutet, S. & Schlichting, I. (2015). *J. Synchrotron Rad.* **22**, 225–238. 10.1107/S1600577515002349 25723924

[bb33] Neutze, R., Wouts, R., van der Spoel, D., Weckert, E. & Hajdu, J. (2000). *Nature (London)*, **406**, 752–757. 10.1038/35021099 10963603

[bb34] Newstead, S., Ferrandon, S. & Iwata, S. (2008). *Protein Sci.* **17**, 466–472. 10.1110/ps.073263108 PMC2248303 18218713

[bb35] Nogly, P., James, D., Wang, D., White, T. A., Zatsepin, N., Shilova, A., Nelson, G., Liu, H., Johansson, L., Heymann, M., Jaeger, K., Metz, M., Wickstrand, C., Wu, W., Båth, P., Berntsen, P., Oberthuer, D., Panneels, V., Cherezov, V., Chapman, H., Schertler, G., Neutze, R., Spence, J., Moraes, I., Burghammer, M., Standfuss, J. & Weierstall, U. (2015). *IUCrJ*, **2**, 168–176. 10.1107/S2052252514026487 PMC4392771 25866654

[bb36] Nogly, P., Weinert, T., James, D., Carbajo, S., Ozerov, D., Furrer, A., Gashi, D., Borin, V., Skopintsev, P., Jaeger, K., Nass, K., Bath, P., Bosman, R., Koglin, J., Seaberg, M., Lane, T., Kekilli, D., Brunle, S., Tanaka, T., Wu, W., Milne, C., White, T., Barty, A., Weierstall, U., Panneels, V., Nango, E., Iwata, S., Hunter, M., Schapiro, I., Schertler, G., Neutze, R. & Standfuss, J. (2018). *Science*, **361**, eaat0094. 10.1126/science.aat0094 29903883

[bb37] Royant, A., Nollert, P., Edman, K., Neutze, R., Landau, E. M., Pebay-Peyroula, E. & Navarro, J. (2001). *Proc. Natl Acad. Sci. USA*, **98**, 10131–10136. 10.1073/pnas.181203898 PMC56927 11504917

[bb38] Schlichting, I. (2015). *IUCrJ*, **2**, 246–255. 10.1107/S205225251402702X PMC4392417 25866661

[bb39] Spudich, J. L. & Luecke, H. (2002). *Curr. Opin. Struct. Biol.* **12**, 540–546. 10.1016/s0959-440x(02)00359-7 12163079

[bb40] Tenboer, J., Basu, S., Zatsepin, N., Pande, K., Milathianaki, D., Frank, M., Hunter, M., Boutet, S., Williams, G. J., Koglin, J. E., Oberthuer, D., Heymann, M., Kupitz, C., Conrad, C., Coe, J., Roy-Chowdhury, S., Weierstall, U., James, D., Wang, D., Grant, T., Barty, A., Yefanov, O., Scales, J., Gati, C., Seuring, C., Srajer, V., Henning, R., Schwander, P., Fromme, R., Ourmazd, A., Moffat, K., Van Thor, J. J., Spence, J. C. H., Fromme, P., Chapman, H. N. & Schmidt, M. (2014). *Science*, **346**, 1242–1246. 10.1126/science.1259357 PMC4361027 25477465

[bb41] Tiefenbrunn, T., Liu, W., Chen, Y., Katritch, V., Stout, C. D., Fee, J. A. & Cherezov, V. (2011). *PLoS One*, **6**, e22348. 10.1371/journal.pone.0022348 PMC3141039 21814577

[bb42] Weierstall, U., James, D., Wang, C., White, T. A., Wang, D., Liu, W., Spence, J. C. H., Doak, R. B., Nelson, G., Fromme, P., Fromme, R., Grotjohann, I., Kupitz, C., Zatsepin, N. A., Liu, H., Basu, S., Wacker, D., Han, G. W., Katritch, V., Boutet, S., Messerschmidt, M., Williams, G. J., Koglin, J. E., Seibert, M. M., Klinker, M., Gati, C., Shoeman, R. L., Barty, A., Chapman, H. N., Kirian, R. A., Beyerlein, K. R., Stevens, R. C., Li, D., Shah, S. T., Howe, N., Caffrey, M. & Cherezov, V. (2014). *Nature Commun.* **5**, 3309.

[bb43] Weinert, T., Olieric, N., Cheng, R., Brünle, S., James, D., Ozerov, D., Gashi, D., Vera, L., Marsh, M., Jaeger, K., Dworkowski, F., Panepucci, E., Basu, S., Skopintsev, P., Doré, A. S., Geng, T., Cooke, R. M., Liang, M., Prota, A. E., Panneels, V., Nogly, P., Ermler, U., Schertler, G., Hennig, M., Steinmetz, M. O., Wang, M. & Standfuss, J. (2017). *Nature Commun.* **8**, 542. 10.1038/s41467-017-00630-4 PMC5599499 28912485

[bb44] White, T. A., Mariani, V., Brehm, W., Yefanov, O., Barty, A., Beyerlein, K. R., Chervinskii, F., Galli, L., Gati, C., Nakane, T., Tolstikova, A., Yamashita, K., Yoon, C. H., Diederichs, K. & Chapman, H. N. (2016). *J. Appl. Cryst.* **49**, 680–689. 10.1107/S1600576716004751 PMC4815879 27047311

[bb45] Wöhri, A. B., Wahlgren, W. Y., Malmerberg, E., Johansson, L. C., Neutze, R. & Katona, G. (2009). *Biochemistry*, **48**, 9831–9838. 10.1021/bi900545e 19743880

[bb46] Zhang, H., Unal, H., Gati, C., Han, G. W., Liu, W., Zatsepin, N. A., James, D., Wang, D., Nelson, G., Weierstall, U., Sawaya, M. R., Xu, Q., Messerschmidt, M., Williams, G. J., Boutet, S., Yefanov, O. M., White, T. A., Wang, C., Ishchenko, A., Tirupula, K. C., Desnoyer, R., Coe, J., Conrad, C. E., Fromme, P., Stevens, R. C., Katritch, V., Karnik, S. S. & Cherezov, V. (2015). *Cell*, **161**, 833–844. 10.1016/j.cell.2015.04.011 PMC4427029 25913193

